# Supplementary material for: Bacterial capture efficiency in fluid bloodstream improved by bendable nanowires
Source: Nat Commun. 2018 Feb 6;9:444. doi: 10.1038/s41467-018-02879-9 (PMC5802748; doi:10.1038/s41467-018-02879-9)
Supplement: Supplementary file 1 — Supplementary information [file 41467_2018_2879_MOESM1_ESM.doc]

**Supplementary Methods**

**Materials**

Melamine foam (MF) was purchased from a local supermarket. The *Salmonella* bacteria strain was from the laboratory of Dr. Baowei Yang, Northwest A&F University (Yangling, Shaanxi, China). Healthy human blood was obtained from Peking University School and Hospital of Stomatology. All chemicals were purchased from Sigma–Aldrich (St. Louis, MO, USA) and used as received without further purification unless otherwise noted. Deionized water (18.25 MΩ·cm) was used throughout.

**Materials characterization** The morphology of the samples was characterized by field-emission scanning electron microscopy (FE-SEM; S-4800; Hitachi, Tokyo, Japan) and transmission electron microscopy (TEM; JEM-1011; JEOL, Tokyo, Japan). The high-resolution TEM (HRTEM) images were recorded by a JEM-2100F microscope (JEOL). The crystalline structures of the samples were characterized using a PANalytical X-ray diffraction (XRD) instrument with Cu Kα radiation ranging from 5 to 70° at room temperature. Thermogravimetric analysis (TGA) experiments were performed using a TG/DTA 6300 instrument (Perkin-Elmer, Wellesley, MA, USA). For NiCo(OH)_2_CO_3_ NWs and NiCo_2_O_4_ NWs preparation, air was flowed through the TGA at 20 mL min^-1^, as the temperature was increased from 25 to 800°C at a heating rate of 10°C min^-1^.

**Atomic Force Microscopy (AFM)** Quantitative mechanical property mapping was carried out using a Dimension FastScan AFM (Bruker AXS Corporation, Santa Barbara, CA, USA), operated under the peak-force taping mode (PeakForce QNM), in which the applied force was 50 nN and the scan rate was set at 1 Hz. The spring constant of the cantilever was 16 N m^-1^, as determined by measuring the free resonance frequency in air. The radius of the standard AFM probe was 8 nm. The spatial distribution of the Young's modulus across the nanowire surface was determined from the AFM load–displacement curves by applying the Derjaguin‒Muller‒Toporov (DMT) model. The cantilever spring constant and sensitivity were calibrated before and after each experiment. Data processing was performed using the commercial Nanoscope Analysis software (Bruker AXS Corporation).

**NW bending test** The single-crystalline NiCo(OH)_2_CO_3_ NWs and polycrystalline NiCo_2_O_4_ NWs were grown on CF, as described above, and introduced into the sample chamber of an environmental scanning electron microscope (ESEM; FEI Quanta 200 FEG; Holland FEI Company, Eindhoven, Holland) equipped with an Omniprobe micromanipulator. For testing of the bending behavior, single NiCo(OH)_2_CO_3_ NWs and NiCo_2_O_4_ NWs were bent by contacting their top with the tungsten tip of the Omniprobe micromanipulator, and deflecting them by linear movement of the tip while recording the deflection by continuous ESEM imaging.

**Description of FEM simulations**

The finite element method (FEM) environment (COMSOL Multiphysics; ver. 5.2) was used to test the proposed explanation for the bending of the NWs. Considering the actual shape of an NW, it was modeled as a truncated cone with a head diameter of 160 nm, root diameter of 15 nm and height of 5 μm, in accordance with TEM and SEM characterizations. Based on the experimental observation that the Young's modulus of the NWs depended on their diameter, and that the degree of NW bending was rather large, the NWs that we simulated were non-linearly elastic[^1^](#_ENREF_1) and incompressible, and could be restored to their initial straight shape without external force. This behavior satisfied the characteristics of hypertonic materials. Herein, we chose the St. Venant–Kirchhoff hyperelastic model to describe this behavior. Full nonlinear displacements of the St. Venant–Kirchhoff material can be used for large displacement calculations when the NW undergoes only small strains. Moreover, it was assumed that a function of elastic energy existed that served as a scalar function for the strain tensor according to the following equation:

$W\text{(}\mathbf{E}\text{)= }\frac{\lambda}{2}\left(\text{tr}\mathbf{E}\right)^{2}+\mu\text{tr}\mathbf{E}^{2}$ (1)

where **E** is the green strain tensor, and *λ* and *μ* are the Lamé constants. The conversion relationships from the Young's modulus (*E*) and Poisson’s ratio (*ν*) to the Lamé constants have the forms:

$\lambda=\frac{E\nu}{(1+\nu)(1-2\nu)}$ (2)

$\mu=\frac{E}{2(1+\nu)}$ (3)

Then, the second Piola–Kirchhoff stress tensor is easily derived as:

$\mathbf{S}(E)=\frac{\partial W(E)}{\partial E}=\lambda(\text{tr}E)I+2\mu E$ (4)

To derive the deformation, we must return to the strain tensor, which is a measure of deformation that represents the displacement between specific points in the body relative to their reference length. Generally speaking, the deformation of a body can be expressed in the form **u** = **F**(**U_0_**) where **U_0_** is the reference position of material points in the body. Then, the strain tensor follows Equation 5:

$E=\frac{\partial(U-U_{0})}{\partial U_{0}}$ (5)

The relationship between the Young's modulus (*E*, GPa) and the diameter (*d*, nm) of two NWs was fitted from the AFM results:

For NiCo(OH)_2_CO_3_ NWs,

$E=E_{0}+A_{1}\text{e}^{\left(d\text{-}x_{0}\right)\text{/}t_{1}}+A_{2}\text{e}^{\left(d\text{-}x_{0}\right)\text{/}t_{2}}$ (6)

where the values of the fitting parameters are *E*_0_ (–36.95), *x*_0_ (–380.49), *A*_1_ (21.31), *A*_2_ (7.06 × 10^-10^), *t*_1_ (674.25) and *t*_2_ (23.56).

For NiCo_2_O_4_ NWs,

$E=\frac{A_{1}-A_{2}}{1+\left(d/d_{0}\right)^{p}}+A_{2}$ (7)

where the values of the fitting parameters are *A*_1_ (0.59), *A*_2_ (31.05), *d*_0_ (140945.91) and *p* (0.54).

The design of the fluid structure interaction (FSI)-based structure includes defining the variables for the required geometry and selection of the parameters. The two-dimensional (2D) model was constructed in the drawing mode of COMSOL Multiphysics. For this, a horizontal micro flow channel 20 μm high and 10 μm long was constructed for the experimental analysis, in which a vertically structured NW (having the dimensions described previously) was placed in the middle of the flow channel. Fluid was allowed to flow from the left side of the geometry to the outlet located at the right side of the flow channel. The Reynolds number of the flow was small (*Re* << 100) due to the small dimensions of the channel, and the flow remained laminar in most of the area. The swirls were restricted to a small area behind the structure. The amount of deformation and size and location of the swirls depended on the magnitude of the inflow velocity. In this study, the simulations were performed using the 2D incompressible Navier–Stokes module under the microfluidics model of COMSOL Multiphysics, which is designed specifically to support the numerical modeling of fluid flow through a channel. Fluid flow in the channel follows the Navier–Stokes equations[^2^](#_ENREF_2). The simulation comprised the application of changing the flow velocity and obstacle direction.

Concerning solid mechanics, to realize the defined system, the bottom of the NW was fixed in space, which corresponded to contact between the CF and the NW; the four side faces were proscribed a displacement. To obtain the von Mises stress of two NWs, the constant displacements were 100 nm and 1 μm for the NiCo(OH)_2_CO_3_ NW and NiCo_2_O_4_ NW, respectively, which were measured from experimental SEM images using ImageJ software (NIH, Bethesda, MD, USA). The entire simulation was time-dependent.

**Surface modification with Con A**

To bind Con A onto the substrate, the NiCo(OH)_2_CO_3_/CF and NiCo_2_O_4_/CF substrates were first treated with oxygen plasma for 30 min (EXTRON25). The smooth CF substrate was immersed in Piranha solution [H_2_SO_4_:H_2_O = 7:3 (v/v)] at 90°C for 2 h to obtain a clean surface with hydroxyl groups. Then, the CF was washed several times with deionized water and finally dried in an argon atmosphere. The treated substrate surface was first incubated in 4% (v/v) 3-aminopropyltriethocysilane (APTES) in ethanol at room temperature for 60 min. Then, the APTES-modified substrates were immersed in a 10 mM bis(N-succinimidyl) carbonate (DSC) solution in acetonitrile at room temperature for 10 min. After the solution was removed, the surface was washed with acetonitrile and dried in an argon atmosphere. Finally, the substrate was treated with 50 μg mL^-1^ bacteria-binding molecule solutions of Con A in phosphate-buffered saline (PBS; 20 mM, pH 7.2) at room temperature for 60 min. After the solution was removed, the substrate was immersed in 5 mL of PBS buffer and shaken for 10 min on a shaker to remove non-covalently attached Con A. After the PBS washing buffer was removed, the substrate modified with Con A was obtained, and was stored at 4°C for later use.

**Surface modification with polyethyleneimine (PEI)**

To graft PEI (branched, MW 1,800 Da) onto substrates, the APTES-modified substrates obtained from the above-mentioned process were incubated in 10 mL of anhydrous acetonitrile solution containing 0.51 g of 4-nitrophenyl chloroformate (NPC) and 0.36 mL of triethylamine (TEA). The reaction was performed at room temperature in an argon atmosphere for an additional 2 h. Then the NPC-activated substrates were cleaned with acetonitrile, deionized water, and acetone and dried in an argon atmosphere. Finally, PEI was grafted to the surface of the substrates by immersing the samples in an aqueous solution containing PEI (5 mg mL^-1^) at room temperature for 2 h. The PEI-modified surfaces were washed with abundant deionized water and acetone to remove the unreacted PEI, and dried in an argon atmosphere.

**Evidence of surface modification with Con A**

We used rhodamine B-labeled Con A (Con A-Rhb) as the detector to assess Con A binding to the substrate. Con A-Rhb was prepared by the protocol provided by the manufacturer of the fluorescence dye, as follows: Rhb was dissolved in anhydrous dimethyl sulfoxide (DMSO) to obtain a 10 mg/mL stock solution. Then, 50 μL of the dye solution was added gradually to 2 mL of Con A solution (50 μg mL^-1^, pH 7.2, phosphate buffer, 20 mM). The reactions were carried out overnight at 4°C. Labeled proteins were then dialyzed against phosphate buffer (20 mM, pH = 7.2) until there was no fluorescence at 555 nm in the dialysate, which was then stored at 4°C for further use. Confocal microscopy images were recorded with an FV1000-IX81 instrument (Olympus, Tokyo, Japan).

**Bacterial culture**

Typically, the *Salmonella* bacteria strain were stored at –80°C in glycerol/Difco nutrient (20% v/v) and reactivated by inoculating the bacteria in 50 mL of sterile Luria–Bertani broth medium at 37°C. After shaking overnight, bacteria cells were harvested by centrifuging (5,000 rpm for 5 min) and washing three times with sterile normal saline. Then, the cells were resuspended in sterile normal saline and diluted to predetermined volumes as stock solutions. The bacterial concentration could be monitored photometrically by measuring the optical density (OD) at a wavelength of 600 nm. Before performing the bacterial capture experiments, the OD_600_ values of the bacteria stock solutions were re-adjusted to 0.1, which corresponded to the concentration of ~10^8^ CFU mL^-1^. The resulting solution was then diluted twice (1:10) to obtain 100 mL of a 10^-2^ stock solution of bacteria for filtering. The solutions before and after filtering were quantified using the standard plate count method in an appropriate culturing agar medium.

**Bacteria morphology**

The morphology of bacteria captured on a substrate was examined by field-emission scanning electron microscopy (FE-SEM; S-4800; Hitachi). After capture, the substrate with bacteria was first fixed with 2.5% glutaraldehyde for 4 h at room temperature. Then, the substrate was washed with sterile normal saline followed by dehydration in increasing concentrations of ethanol (25, 50, 75, 90, 95, 100%) for 10 min with each step, and dried in air overnight. Prior to imaging, the bacteria on a substrate were sputter-coated with platinum and imaged by FE-SEM.

**Statistical analysis** Captured bacteria numbers are reported as means ± standard deviations (sd) from three independent experiments. Significance was calculated using a two-way analysis of variance with Tukey's post hoc test using Minitab software (Minitab, Inc., State College, PA, USA). P values < 0.05 were considered statistically significant with ^*^P < 0.05 and ^**^P < 0.01. P values > 0.05 were considered non-significant (ns).

**Supplementary Figures**

**
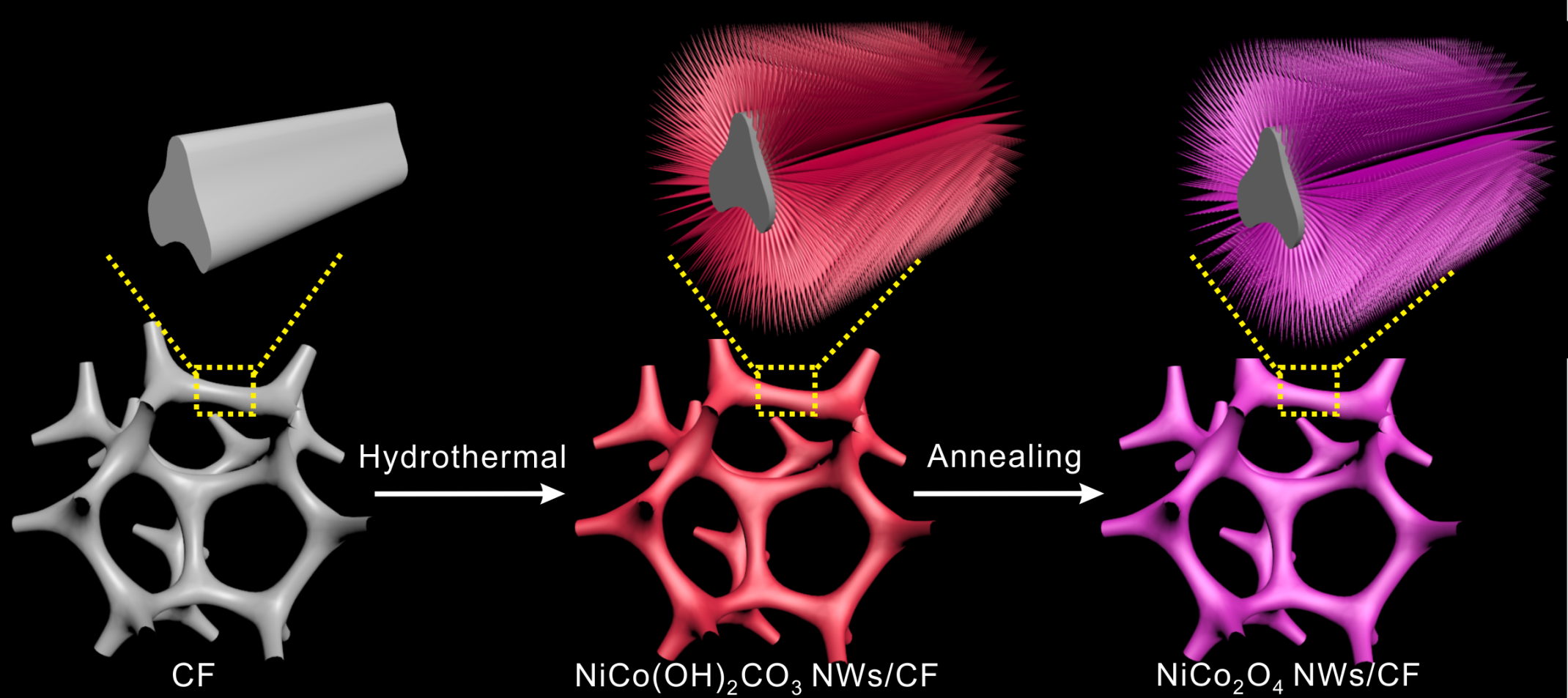
**

**Supplementary Figure 1** Schematic illustration of the formation of dialyzer. The NiCo(OH)_2_CO_3_ nanowires(NWs)/carbon foam(CF) and NiCo_2_O_4_ NWs/CF composite are fabricated by hydrothermal and annealing process.


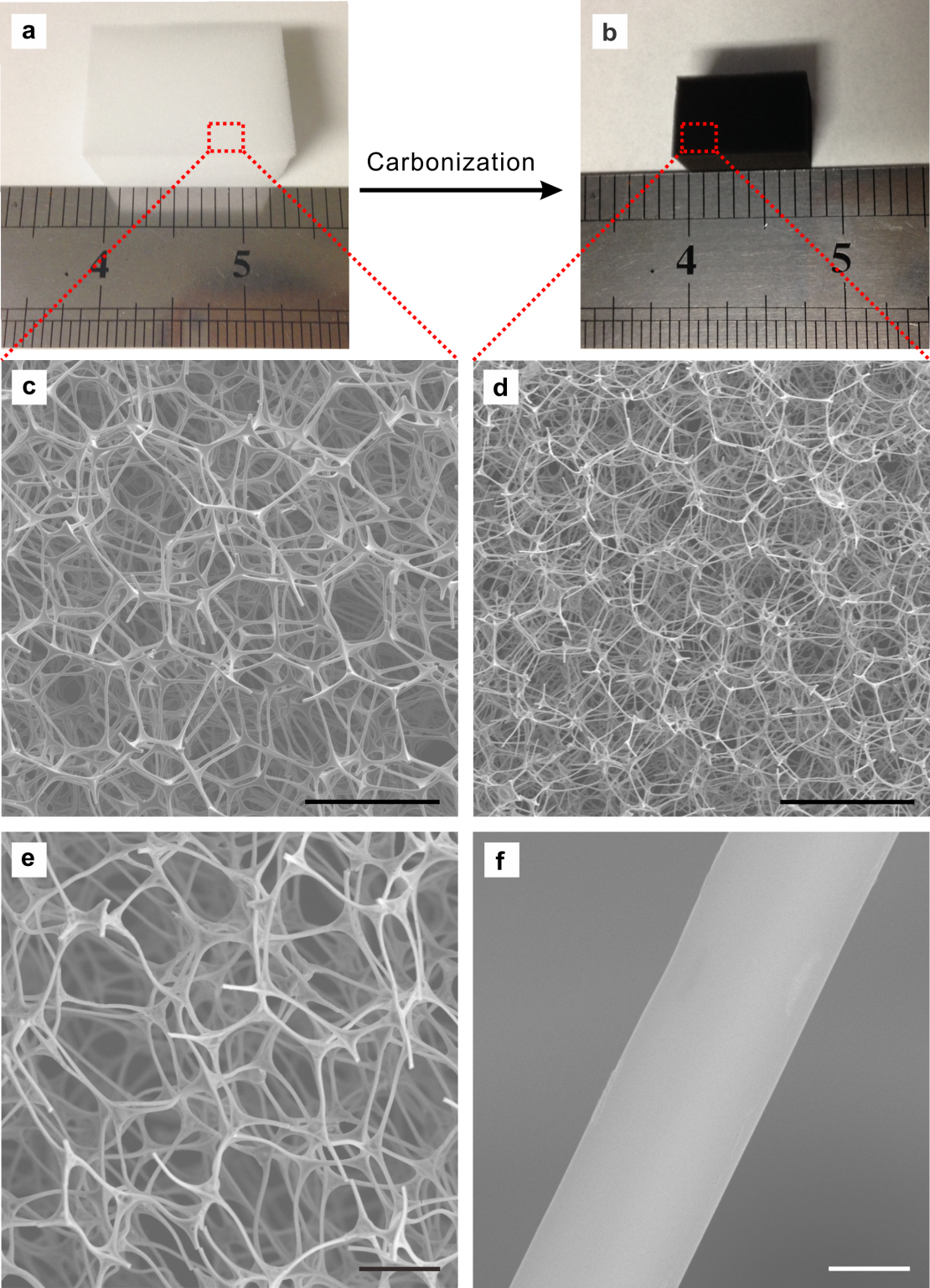


**Supplementary Figure 2** Morphology characterization of CF. Digital photos of (**a**) original melamine foam (MF) and (**b**) CF after carbonization process. Low SEM images of (**c**) MF and (**d**) CF. High magnification SEM images of (**e**) CF and (**f**) one network fiber in the CF. Scale bars in (**c**) and (**d**) are 250 μm. Scale bar in **(e**) is 50 μm. Scale bar in **(f**) is 2 μm.


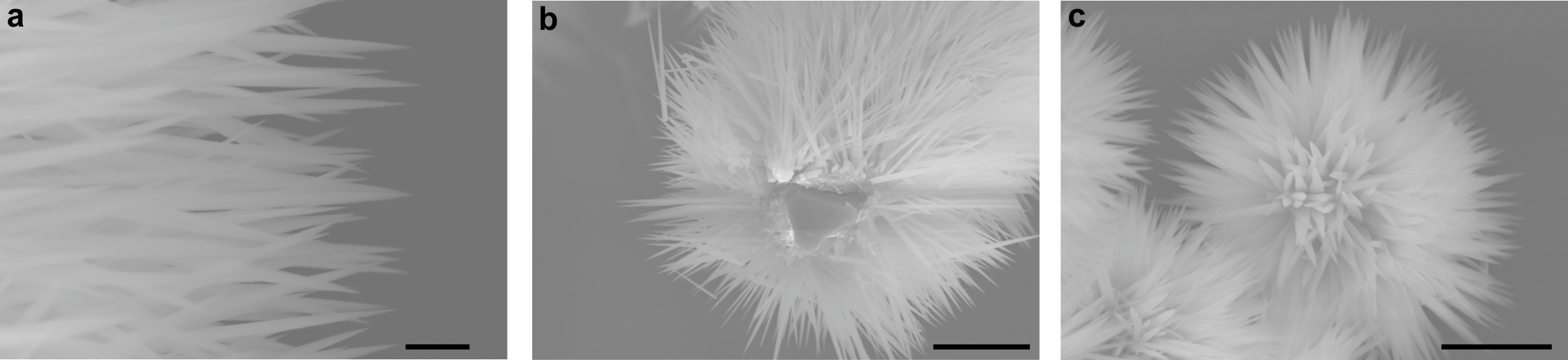


**Supplementary Figure 3** SEM images of NiCo(OH)_2_CO_3_ NWs. Representative SEM images of NiCo(OH)_2_CO_3_ NWs arrays grown CF with (**a**) side view and (**b**) cross section view. (**c**) SEM image of NiCo(OH)_2_CO_3_ NWs microsphere prepared in the absence of CF. Scale bar in (**a**) is 500 nm. Scale bar in (**b**) is 5 μm. Scale bar in (**c**) is 2 μm.


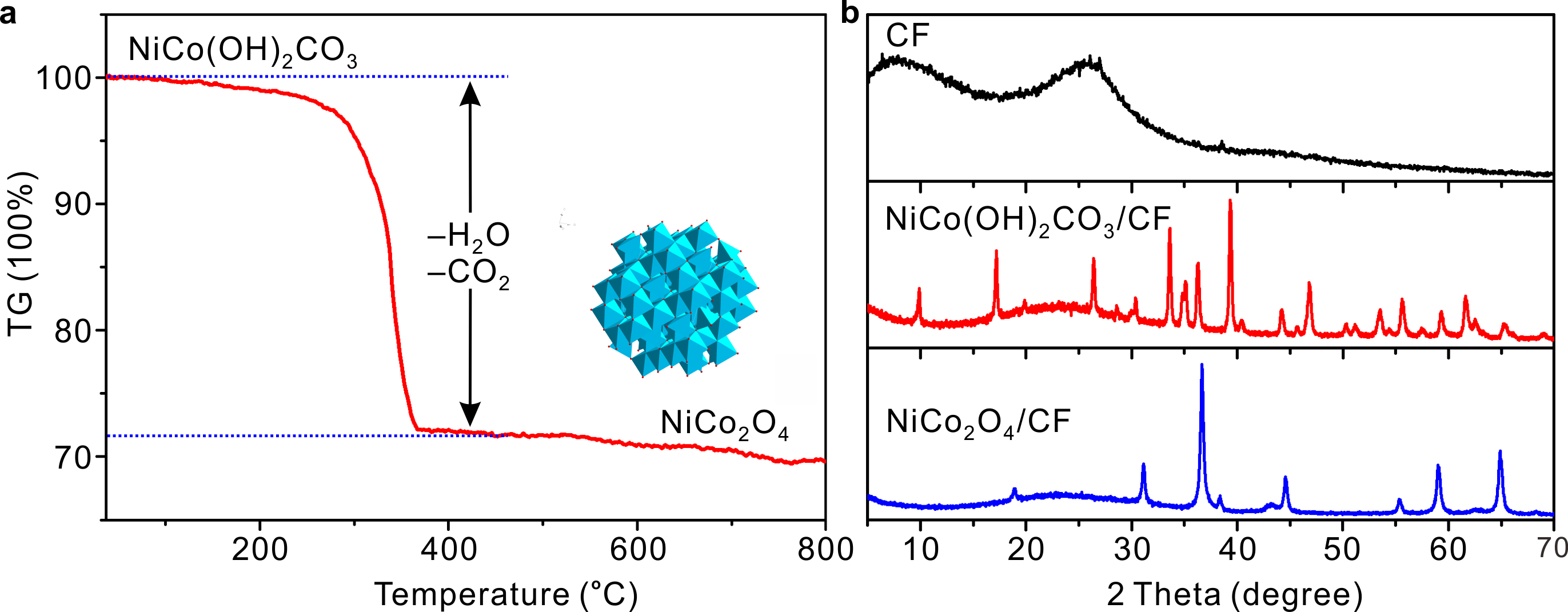


**Supplementary Figure 4** Structural characterization of NWs. (**a**) The TGA analysis results of the as-prepared NiCo(OH)_2_CO_3_ NWs. (**b**) XRD patterns of CF, NiCo(OH)_2_CO_3_/CF and NiCo_2_O_4_/CF.


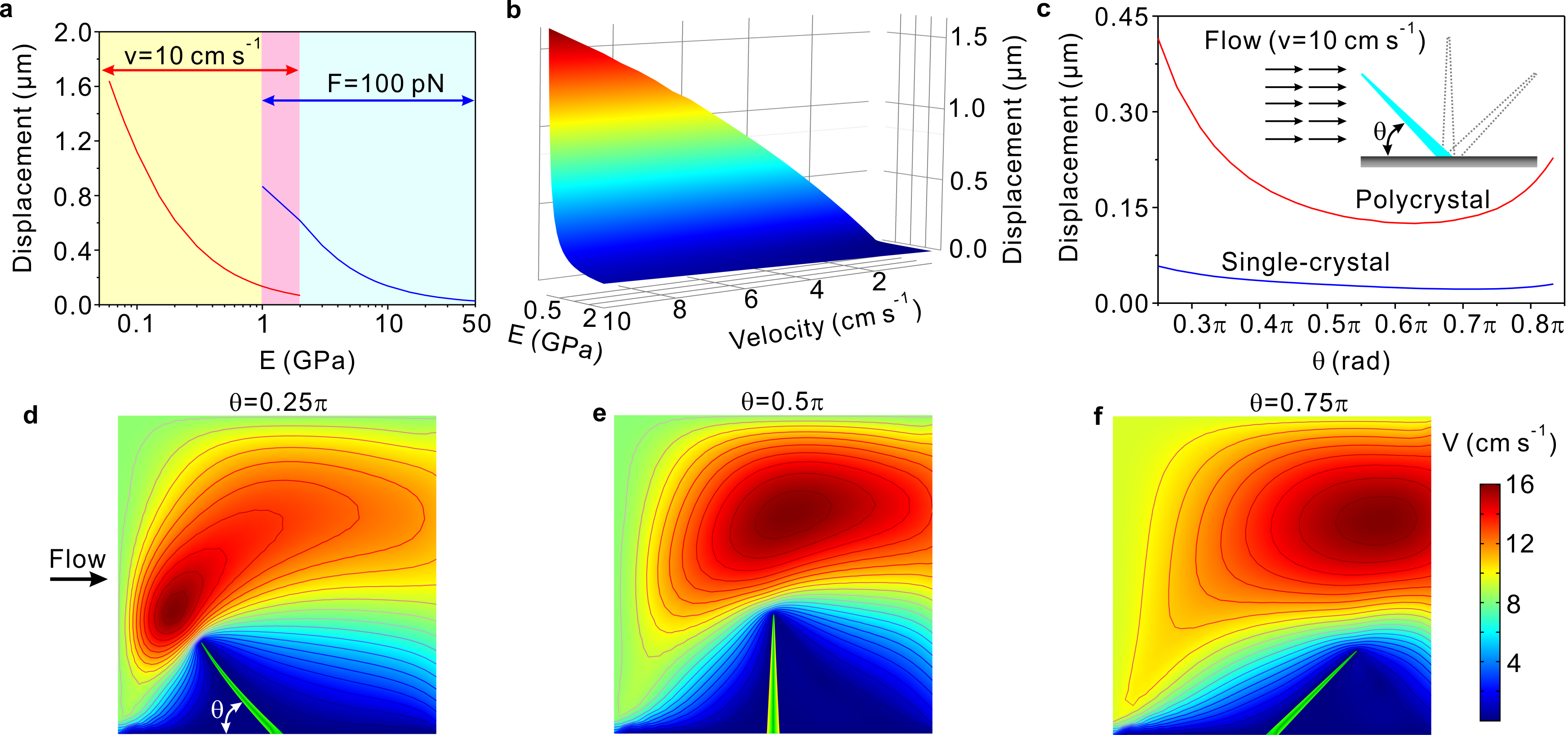


**Supplementary Figure 5** Fluid dynamics analysis of NWs. (**a**) The relationship between Young’s modulus (E) and the lateral deflection distance (*x* displacement) under the high flow velocity (v=10 cm s^-1^) and the applied parallel force (F=100 pN), respectively, determined by FEM. (**b**) A 3D surface plot shows the effect of E and flow velocity on the displacement of a single NW. (**c**) The deformation of single-crystalline and polycrystalline NWs calculated at different angles (θ), with a schematic of the orientation of the NWs and flow velocity (v=10 cm s^-1^) shown in the inset. (**d**-**f**) The flow field around the single NW and the NW deformation result with three kinds of angles (θ): θ=0.25π (**d**), θ=0. 5π (**e**) and θ=0.75π (**f**).


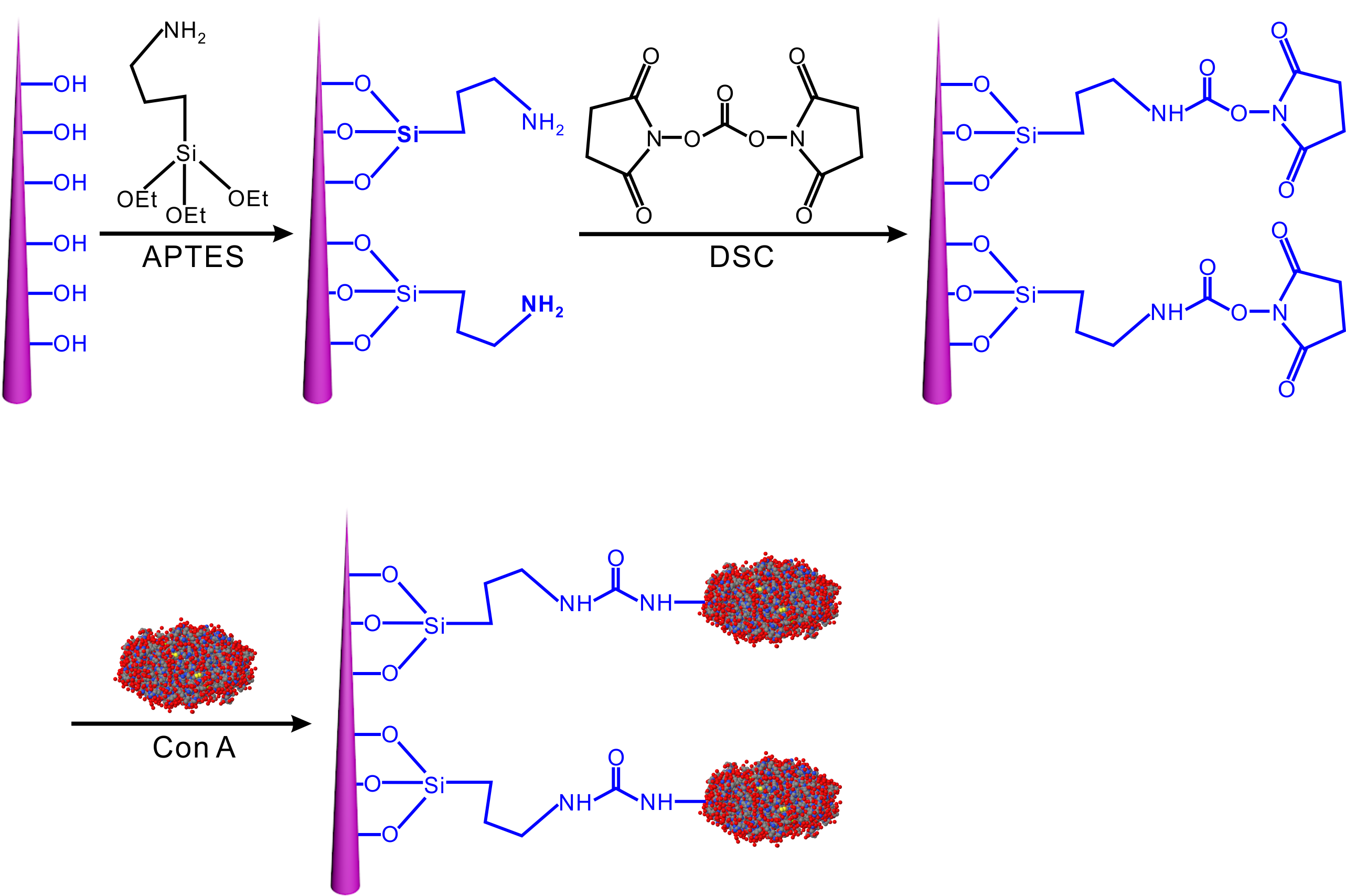


**Supplementary Figure 6** The progress of surface functionalization. Chemical modification of the NWs (including CF) with 3-aminopropyltriethocysilane (APTES), bis(N-succinimidyl) carbonate (DSC), and Concanavalin A (Con A), respectively.


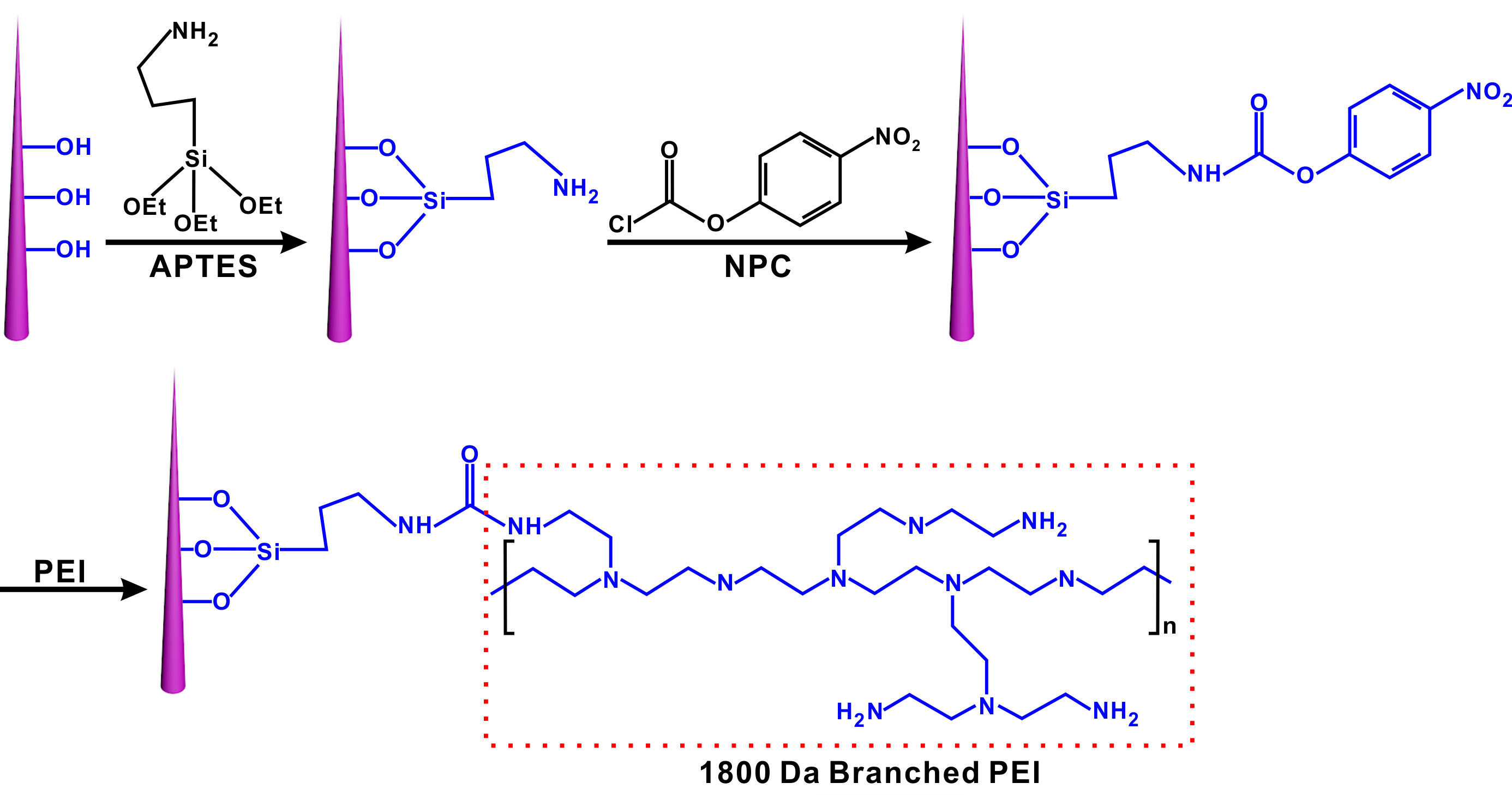


**Supplementary Figure 7** NW surface of positive charge. Scheme of the NWs surface modification with 3-aminopropyltriethocysilane (APTES), 4-nitrophenyl chloroformate (NPC), and 1800 Da branched polyethylenimine (PEI), respectively.


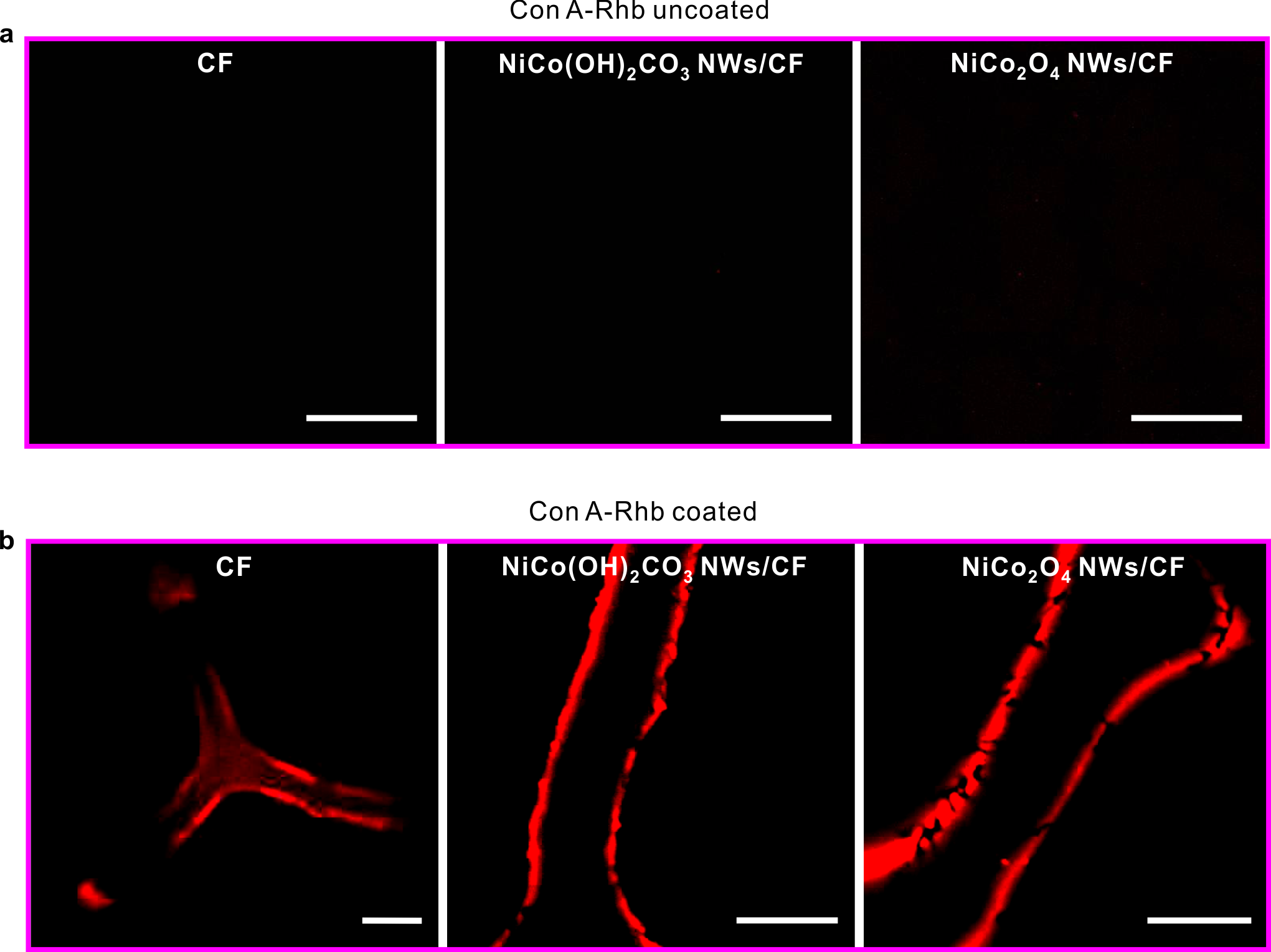


**Supplementary Figure 8** Confirmation of Con A grafted onto NWs. The confocal microscopy fluorescent images of dialyzers with (**a**) Con A-Rhb uncoated and (**b**) Con A-Rhb coated. Scale bars in (**a**) are 100 μm. Scale bars in (**b**) are 20 μm.


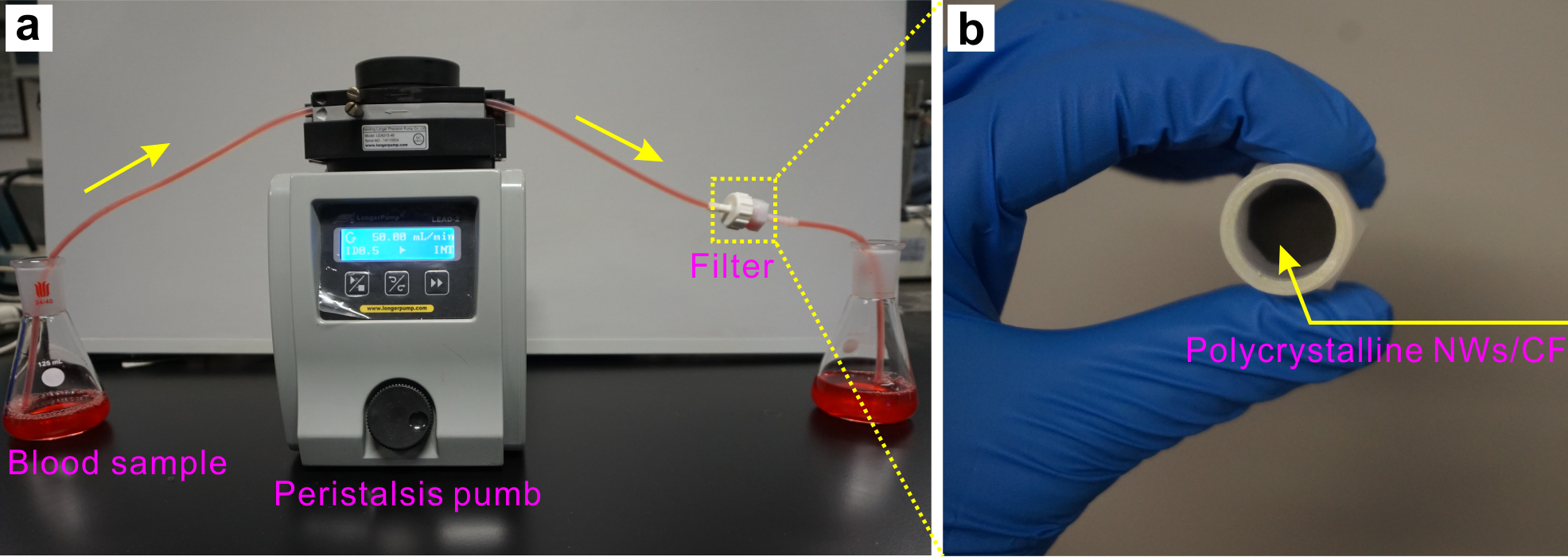


**Supplementary Figure 9** Digital photos of filtration setup and cleansing device. (**a**) Experiment setup showing filtration device during operation. (**b**) Cross-section of cleansing device with substrates into the dialyzer.


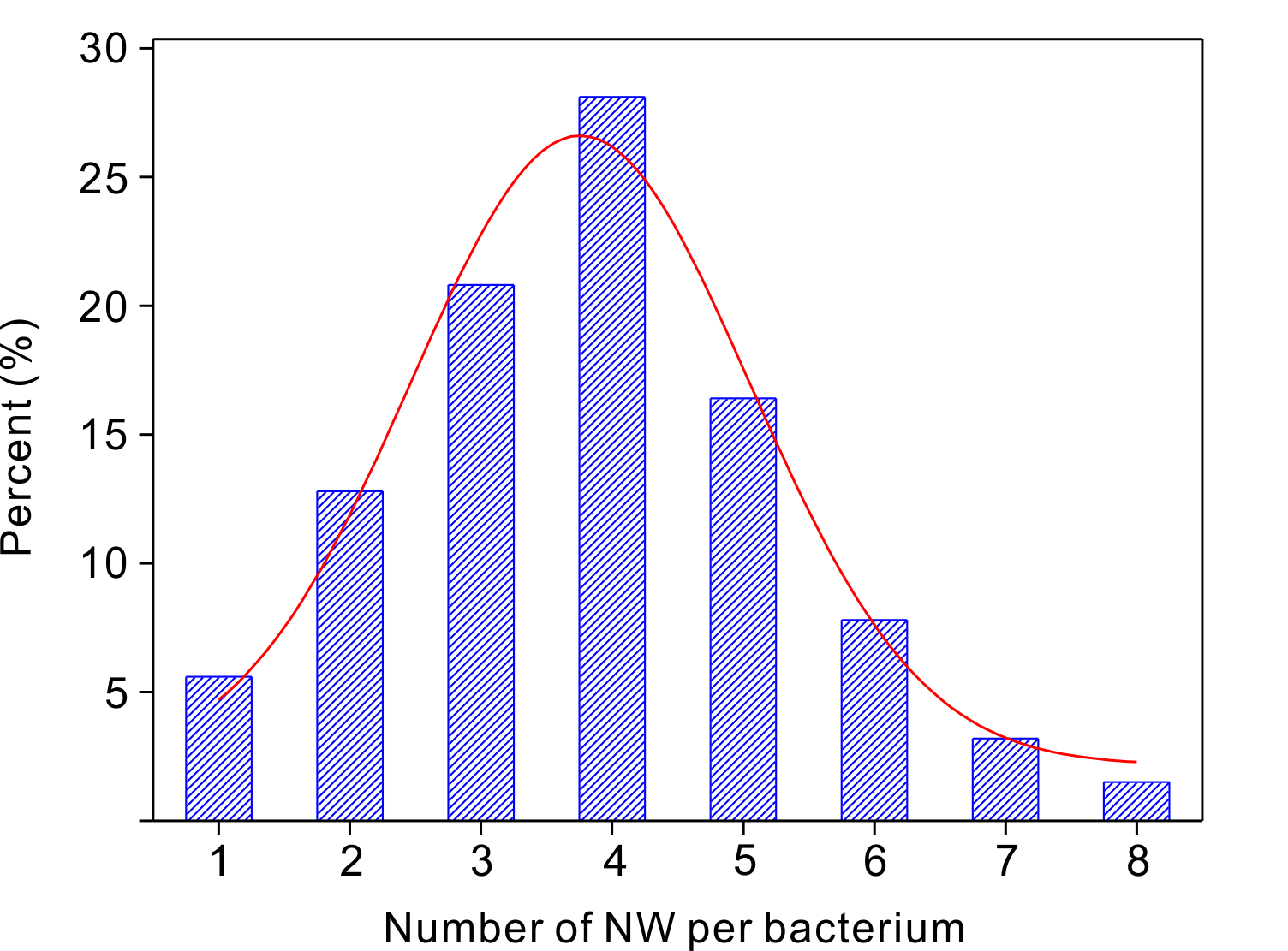


**Supplementary Figure 10** The number of NW required to form a nanoclaw. The distribution of number of NW were collected in histograms. Gaussian (normal) fitting was performed for peak number (solid line).


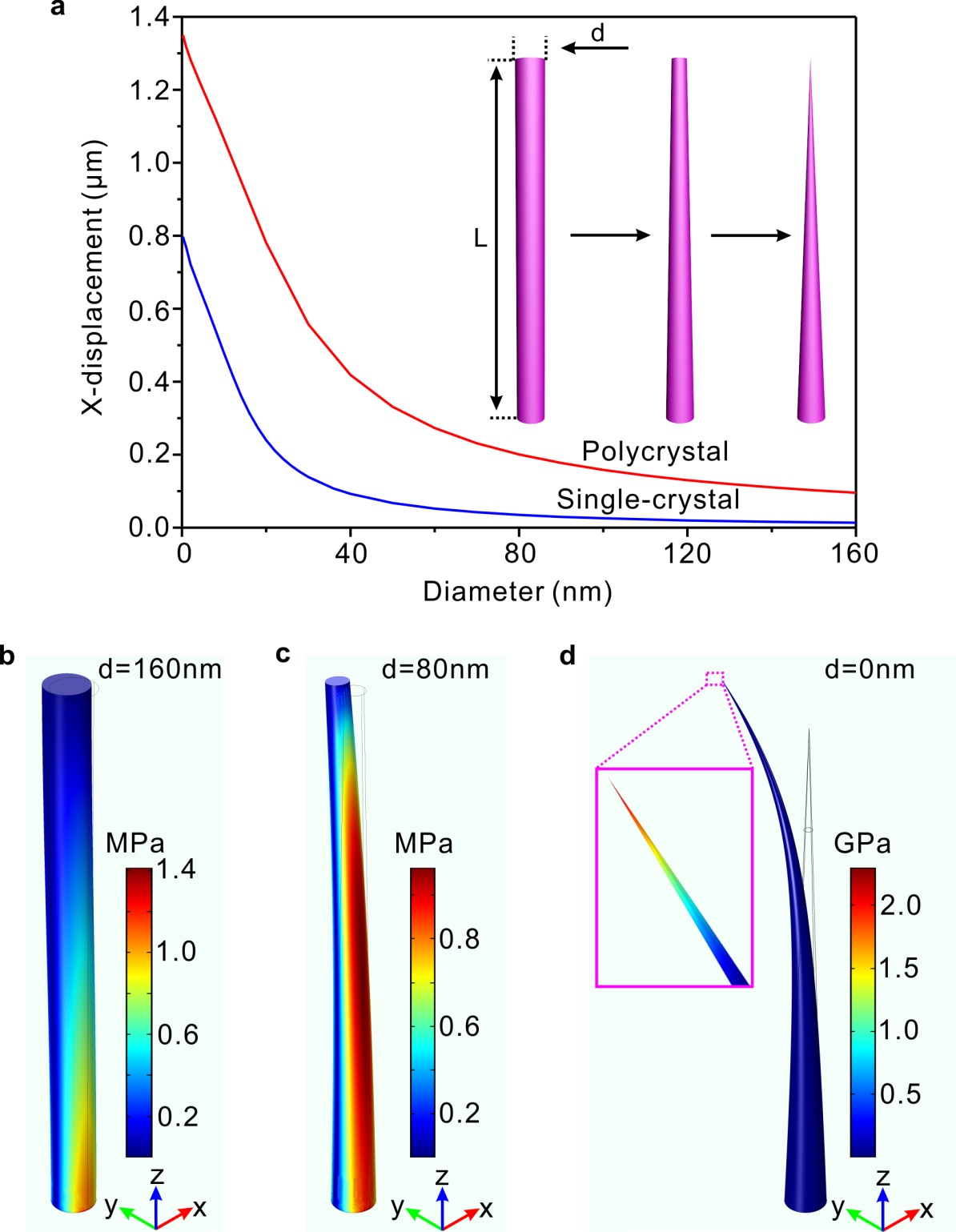


**Supplementary Figure 11** Finite element method (FEM) analysis of NWs bending. (**a**) The relationship between the head diameter (d) of two NWs and the lateral deflection distance (*x* displacement) under a parallel applied force (F=100 pN), determined by FEM. The inset shows that the shape of the NW changes from a cylinder to a cone with decreasing d while maintaining the length (L) of the NW constant. (**b**-**d**) The von Mises stress distribution of polycrystalline NWs at the top, with diameters of 160 (**b**), 80 (**c**), and 0 nm (**d**).


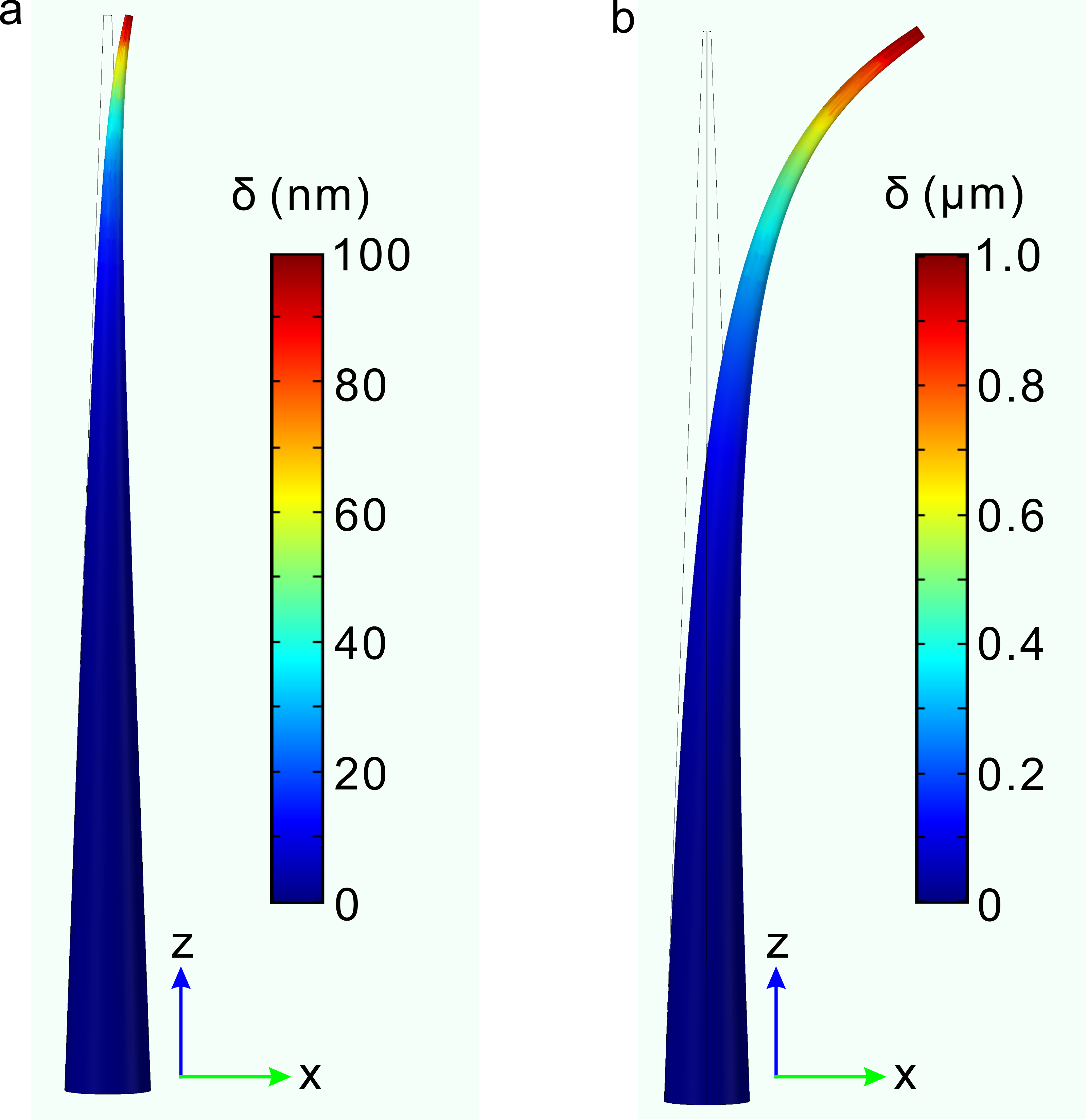


**Supplementary Figure 12** FEM analysis of NW deformation. FEM calculated the deformation of (**a**) single-crystalline NW and (**b**) polycrystalline NW. The color code represents the lateral displacement.


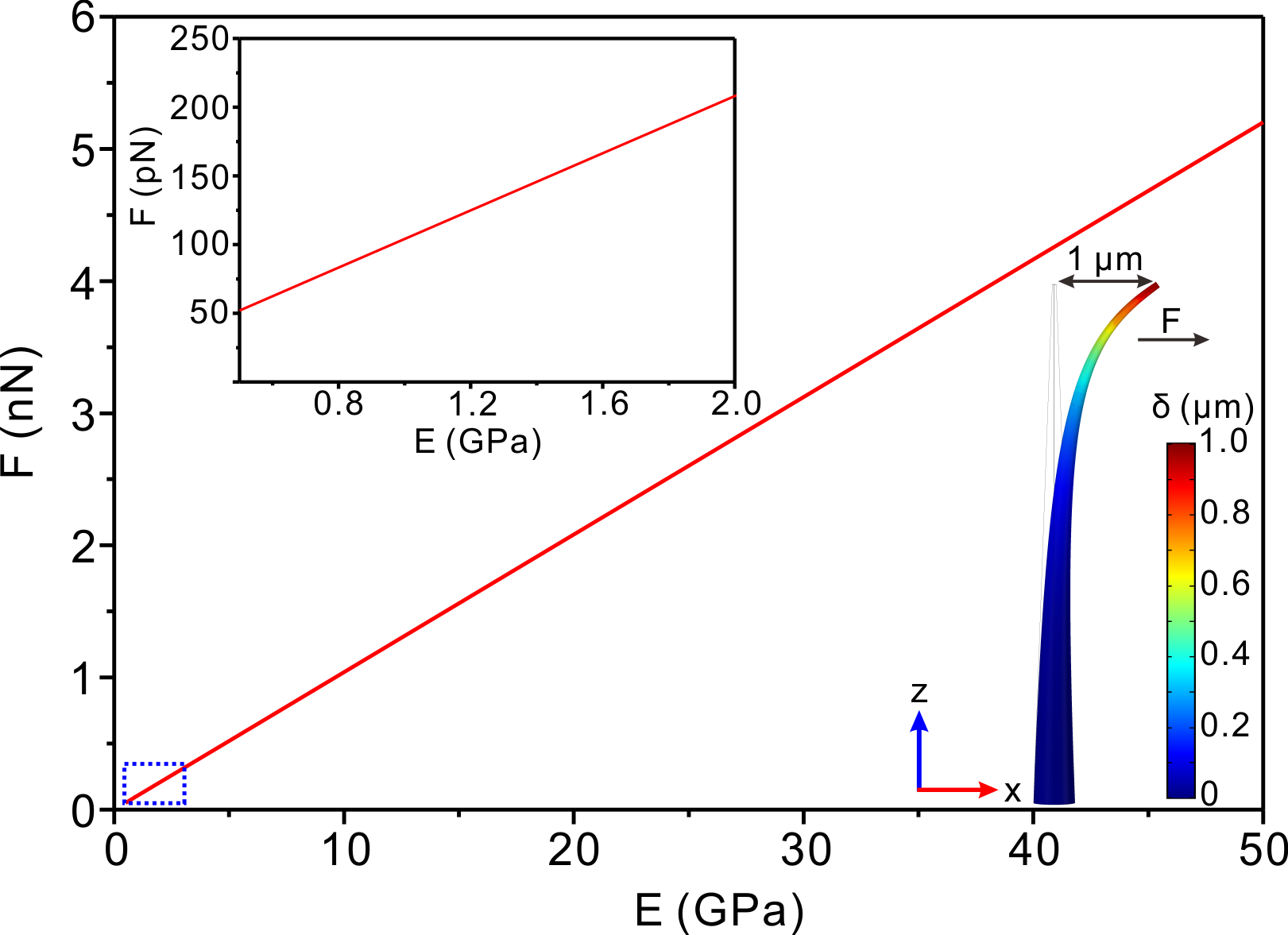


**Supplementary Figure 13** The general condition to form nanoclaws. FEM was used to calculate the relationship between Young’s modulus (E) and the interface force under a constant deflection distance of 1 μm. The Young’s modulus of this single NW was constant for this FEM analysis.


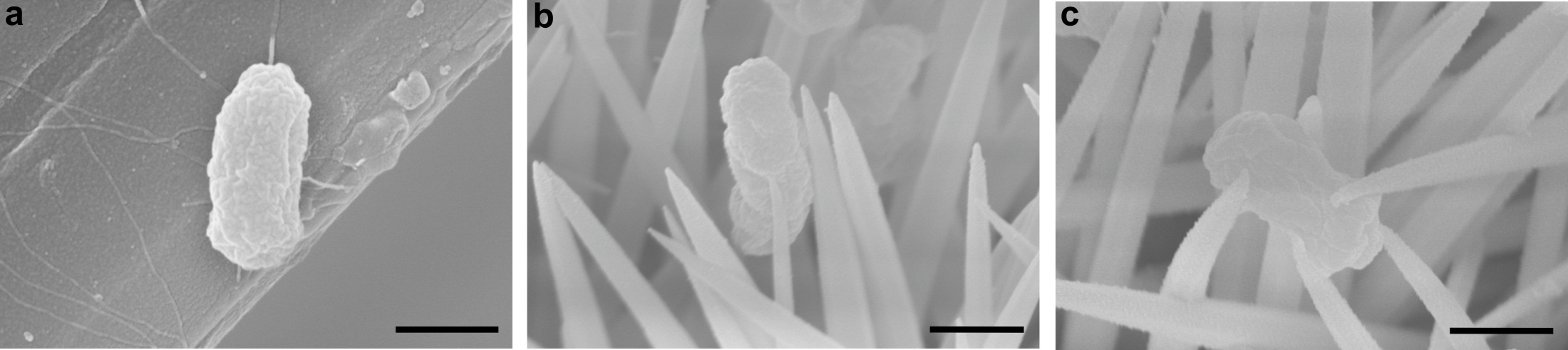


**Supplementary Figure 14** The residual bacteria after washing. SEM images of bacteria captured onto (**a**) CF, (**b**) single-crystalline NWs/CF, (**c**) polycrystalline NWs/CF after washing at high magnification. Scale bars, 500 nm.


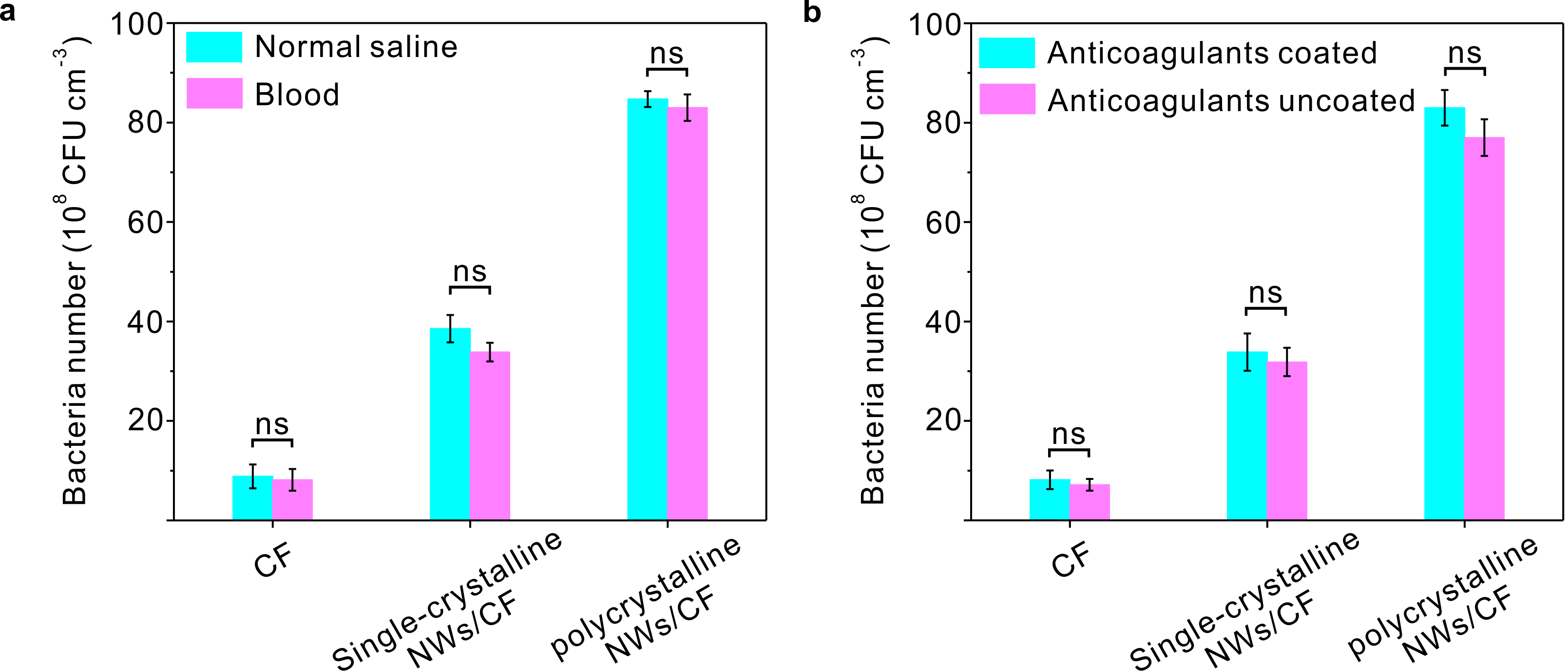


**Supplementary Figure 15 E**ffect of blood cells and anticoagulants on capture efficiency. (**a**) The difference of bacteria-capture number in normal saline and blood for three dialyzers (CF, single-crystalline NWs/CF and polycrystalline NWs/CF). (**b**) The difference of bacteria-capture number in blood samples treated with anticoagulants or not for three dialyzers (CF, single-crystalline NWs/CF and polycrystalline NWs/CF). Error bars: standard error (n=3). Student’s t-test, ns, not significant.


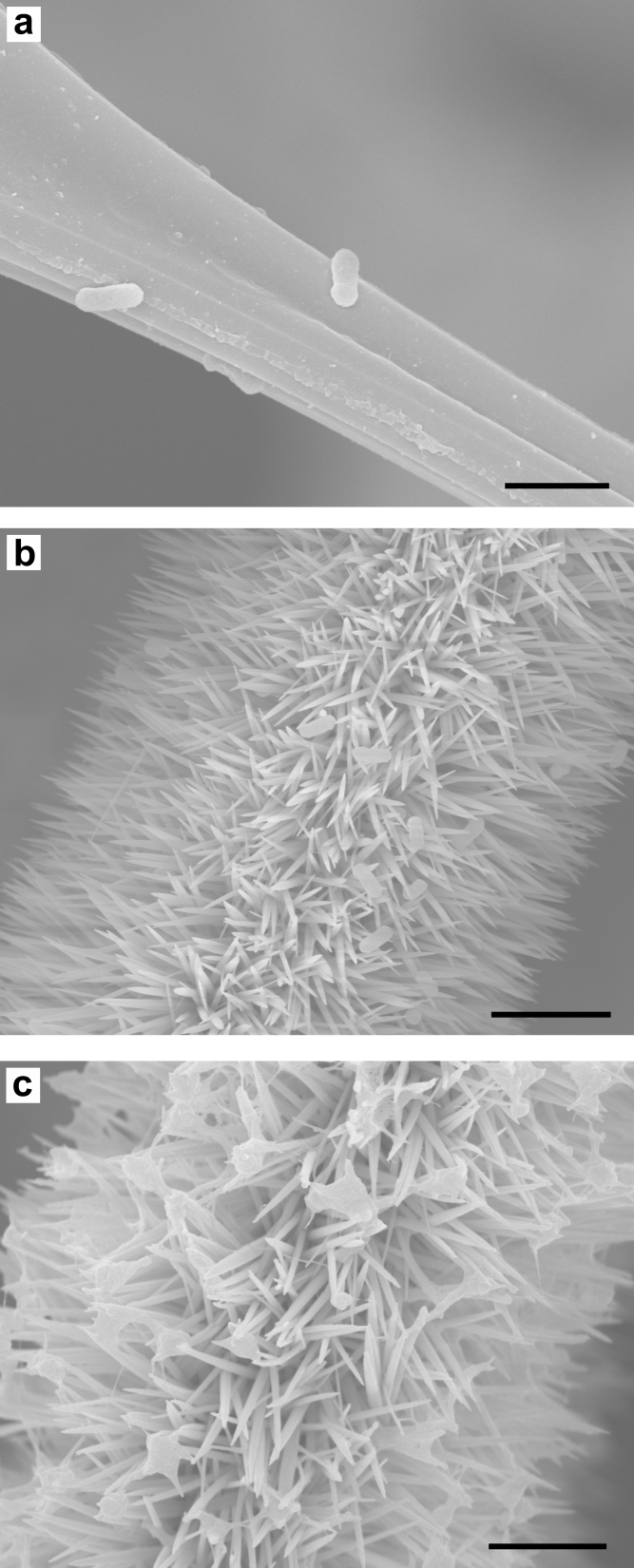


**Supplementary Figure 16** The resudual blood cells on dialyzer. SEM images of (**a**) CF, (**b**) single-crystalline NWs/CF and (**c**) polycrystalline NWs /CF after filtration. Scale bar in (**a**) is 2 μm. Scale bars in (**b)** and (**c**) are 4μm.


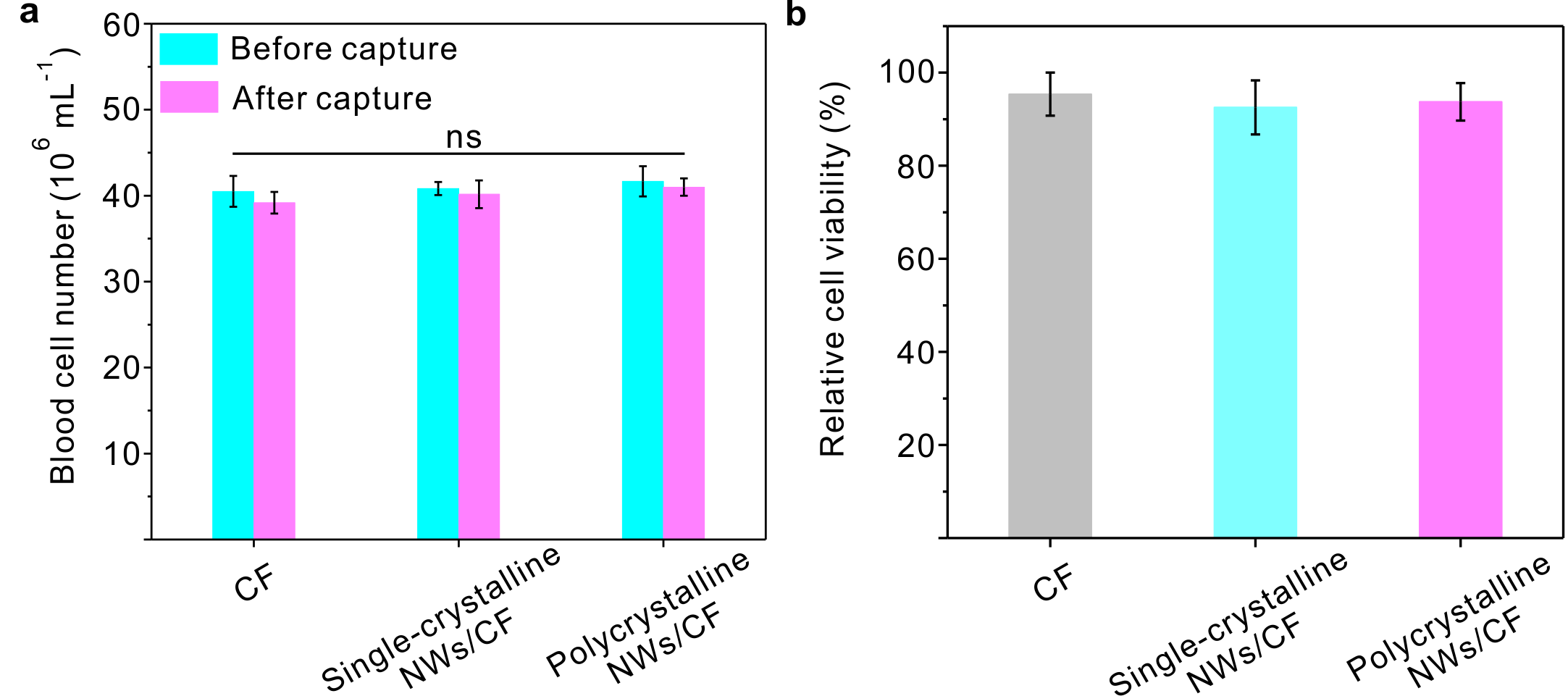


**Supplementary Figure 17** The effect of dialyzer on blood cells. (**a**) The changing of blood cell number after run through the three dialyzers (CF, single-crystalline NWs/CF and polycrystalline NWs/CF). Error bars: standard error (n=3). Student's t-test, ns, not significant. (**b**) The blood cell viability after run through the three dialyzers.

**Supplementary References:**

1. Ogden, R. W. *Non-linear elastic deformations*. 204-222, (Dover Publications, New York, 1997).

2. Bathe, K.-J. & Zhang, H. Finite element developments for general fluid flows with structural interactions. *Int. J. Numer. Meth. Eng*. **60**, 213-232 (2004).
